# Supplementary material for: Unquantifiably low aldosterone concentrations are prevalent in hospitalised COVID-19 patients but may not be revealed by chemiluminescent immunoassay
Source: Endocr Connect. Author manuscript; Available in PMC 2022 Nov 1. (PMC9578067; doi:10.1530/EC-22-0190)

Supplement

eTable 1. Patient characteristics after first positive SARS-CoV-2 test.

Characteristic	All SARS-CoV-2 positive patients	Our cohort	SARS-CoV-2 positive patients not in our cohort	p-value[1]
Sample size (*n*)	1801	134	1667	-
Age at admission, median [IQR] (% missing)	65 [49, 79] (0%)	64 [46, 88] (0%)	66 [49, 79] (0%)	0.24
Gender (male), *n* (%)	973 (54.0%)	80 (59%)	893 (53.6%)	0.20
Ethnicity, n (%)				
White	1254 (69.6%)	90 (67.2%)	1164 (69.8%)	0.58
Black	23 (1.3%)	1 (0.7%)	22 (1.3%)	0.87
Asian	95 (5.3%)	13 (9.7%)	82 (4.9%)	0.029
Other	61 (3.4%)	3 (2.2%)	58 (3.5%)	0.61
Not specified/prefer not to say	368 (20.4%)	27 (20.1%)	341 (20.4%)	~1
Body mass index, median [IQR] (% missing), kg/m	27.3 [23.5, 31.7] (19.8%)	27.8 [23.5, 32.3] (11.9%)	27.3 [23.6, 31.7] (20.4%)	0.32
Observations, median [IQR] (% missing)				
Heart rate, beats/min	85 [74, 96] (11.5%)	86 [74, 95] (7.5%)	88 [80, 99] (11.8%)	0.38

Temperature, °C	37.1 [36.6, 37.7] (11.5%)	37.1 [36.6, 37.7] (7.5%)	37.1 [36.6, 37.8] (11.8%)	0.68
Respiratory rate, breaths/min	18 [17, 21] (11.5%)	19 [17, 22] (7.5%)	18 [17, 21] (11.8%)	0.71
Oxygen saturation (SpO2), %	96 [94, 98] (11.6%)	96 [94, 98] (7.5%)	96 [94, 98] (11.9%)	0.73
Mean arterial pressure, mmHg	89 [80, 99] (11.5%)	90 [82, 99] (7.5%)	88 [81, 100] (11.8%)	0.93
Blood tests, median [IQR] (% missing)				
C-reactive protein, mg/L	43 [14, 101] (1.2%)	51 [19, 107] (1.5%)	43 [14, 100] (1.2%)	0.21
White cell count, 10^9/L	6.4 [4.8, 8.9] (0.5%)	6.2 [5,8.9] (0%)	6.4 [4.8, 8.9] (0.5%)	0.29
Sodium, mmol/L	137.5 [135, 140] (1.1%)	137.8 [135.6, 140] (0%)	137.5 [135, 140] (1.2%)	0.71
Potassium, mmol/L	4 [3.7, 4.3] (1.5%)	4 [3.8, 4.5] (1.5%)	4 [3.7, 4.4] (1.5%)	0.31
Neutrophils, 10^9/L	4.7 [3.3, 7] (0.5%)	4.7 [3.4, 7] (0%)	4.7 [3.3, 6.9] (0.5%)	0.56
Lymphocytes, 10^9/L	0.9 [0.6, 1.4] (0.5%)	1.0 [0.7, 1.5] (0%)	0.9 [0.6, 1.4] (0.5%)	0.21
Interleukin-6, pg/ml	11.2 [3, 30.3] (71.5%)	8.1 [3.6, 18.5] (64.2%)	11.9 [3, 32.4] (72%)	0.011
Urea, mmol/L	6.2 [4.4, 9.4] (5.1%)	5.8 [4.2, 8.8] (0.7%)	6.2 [4.5, 9.4] (5.5%)	0.27
Creatinine, μmol/L	72 [58.5, 93] (0.8%)	72 [62, 93] (0.7%)	72 [58, 93] (0.8%)	0.11

D-Dimer, ng/ml	268 [145, 590] (41.1%)	221 [142, 461] (38.8%)	272 [145, 595] (41.2%)	0.62
Troponin, ng/L	10 [3, 33] (36.9%)	8.5 [3, 24.7] (31.3%)	10.3 [3, 34] (37.3%)	0.82
pH value	7.40 [7.37, 7.43] (6.7%)	7.40 [7.37, 7.44] (3.7%)	7.40 [7.37, 7.43] (7%)	0.53
Past medical history[2], n (%)				
Heart disease	347 (19.3%)	24 (17.9%)	323 (19.4%)	0.76
Hypertension	656 (36.4%)	48 (35.8%)	608 (36.5%)	0.95
Diabetes	389 (21.6%)	44 (32.8%)	345 (20.7%)	0.0015
Endocrine disease (other than diabetes)	164 (9.1%)	19 (14.2%)	145 (8.7%)	0.05
Stroke	68 (3.8%)	3 (2.2%)	65 (3.9%)	0.46
Dementia	151 (8.4%)	10 (7.5%)	141 (8.5%)	0.81
Asthma	244 (13.5%)	20 (14.9%)	224 (13.4%)	0.72
Respiratory disease (other than asthma)	218 (12.1%)	17 (12.7%)	201 (12.1%)	0.94
Chronic kidney disease	141 (7.8%)	9 (6.7%)	132 (7.9%)	0.74
Chronic liver disease	96 (5.4%)	10 (7.5%)	86 (5.2%)	0.35
Malignancy non-haematological	246 (13.7%)	18 (13.4%)	228 (13.7%)	~1
Malignancy	87 (4.8%)	4 (3.0%)	83 (5.0%)	0.41

haematological				
Immunocompromised	13 (0.7%)	2 (1.5%)	11 (0.6%)	0.57
Treatments and outcomes, n (%)				
In-hospital deaths	294 (16.3%)	18 (13.4%)	276 (16.6%)	0.41
Admitted to ICU	331 (18.4%)	15 (11.2%)	316 (18.9%)	0.034
Invasive mechanical ventilation	222 (12.3%)	12 (8.9%)	210 (12.6%)	0.27
Renal replacement therapy	198 (11.0%)	10 (7.5%)	188 (11.3%)	0.22

[1] Mood's test for medians or Chi-squared test for categorical data

[2]See eTable 3 for ICD-10 code lists

[3]Bisoprolol, Atenolol, Propranolol, Carvedilol.

eTable 2. Patient characteristics following the first positive SARS-CoV-2 test, by value of first aldosterone thereafter.

**Characteristic**	**Aldosterone <= 70 pmol/L**	**Aldosterone > 70 pmol/L**	**p-value[1]**
Sample size (*n*)	74	52	
Age at admission, median [IQR] (% missing)	64 [48.25, 82] (0%)	58 [41, 77] (0%)	0.28
Gender (male), *n* (%)	43 (58.1%)	30 (57.7%)	~1
Ethnicity, n (%)			
White	46 (62.2%)	36 (69.2%)	0.529
Black	1 (1.4%)	0 (0%)	~1
Asian	9 (12.2%)	4 (7.7%)	0.607
Other	2 (2.7%)	1 (1.9%)	~1
Not specified/prefer not to say	16 (21.6%)	11 (21.2%)	~1
Body mass index, median [IQR] (% missing), kg/m	25.4 [23.2, 29.6] (9.5%)	28.6 [24.75, 32.85] (9.6%)	0.14
Observations, median [IQR] (% missing)			
Heart rate, beats/min	85 [75, 96] (1.4%)	88 [77, 95] (0%)	0.61
Temperature, ℃	37.4 [36.8, 38.1] (0%)	37.1 [36.7, 37.7] (0%)	0.75
Respiratory rate,	19 [17, 22] (0%)	18 [17, 20] (0%)	0.058

breaths/min			
Oxygen saturation (SpO_2), %	95 [94, 97] (1.4%)	96 [94, 97] (0%)	0.40
Mean arterial pressure, mmHg	90 [80, 100] (0%)	91 [83, 97] (0%)	0.11
Blood tests, median [IQR] (% missing)			
C-reactive protein, mg/L	54 [26, 114] (2.7%)	39 [25, 77] (5.8%)	0.45
White cell count, 10^9/L	5.9 [4.8, 6.8] (2.7%)	5.3 [4.4, 7.1] (1.9%)	0.85
Sodium, mmol/L	136 [134, 139] (4.1%)	137 [134, 139] (5.8%)	0.89
Potassium, mmol/L	4.0 [3.8, 4.3] (1.5%)	4.1 [3.7, 4.6] (1.5%)	0.13
Neutrophils, 10^9/L	4.3 [3.2, 5.4] (2.7%)	3.8 [2.6, 5.5] (1.9%)	0.34
Lymphocytes, 10^9/L	0.84 [0.55, 1.12] (2.7%)	1.07 [0.71, 1.61] (1.9%)	0.44
Interleukin-6, pg/ml	9.5 [5.73, 20.89] (59.5%)	7.15 [3.86, 13.16] (50%)	0.40
Urea, mmol/L	5.4 [3.7, 8.5] (10.8%)	5.9 [4.4, 7.3] (15.4%)	0.56
Creatinine, μmol/L	72 [59, 89] (4.1%)	76 [64, 88] (3.8%)	0.92
D-Dimer, ng/ml	233 [176, 373] (41.9%)	205 [155, 362] (50%)	0.37
Troponin, ng/L	9 [3.1, 32.9] (41.9%)	4 [3, 7.5] (40.4%)	0.95
pH value	7.41 [7.38, 7.44] (39.2%)	7.4 [7.37, 7.44] (30.8%)	0.94
Medical history[2], n (%)			

Heart disease	12 (16.2%)	9 (17.3%)	~1
Hypertension	23 (31.1%)	12 (23.1%)	0.43
Beta blockers at admission[3]	6 (8.1%)	3 (5.75%)	0.88
Diabetes	19 (25.7%)	10 (19.2%)	0.53
Endocrine disease (other than diabetes)	7 (9.5%)	6 (11.5%)	0.94
Stroke	2 (2.7%)	1 (1.9%)	~1
Dementia	5 (6.8%)	2 (3.8%)	0.76
Asthma	6 (8.1%)	8 (15.4%)	0.32
Respiratory disease (other than asthma)	8 (10.8%)	5 (9.6%)	~1
Chronic kidney disease	4 (5.4%)	2 (3.8%)	~1
Chronic liver disease	4 (5.4%)	4 (7.7%)	0.88
Malignancy non-haematological	13 (17.6%)	3 (5.8%)	0.092
Malignancy haematological	4 (5.4%)	0 (0%)	0.24
Immunocompromised	2 (2.7%)	0 (0%)	0.64
Treatments and outcomes, n (%)			
In-hospital deaths	10 (13.5%)	6 (11.5%)	0.96

Admitted to ICU	9 (12.2%)	7 (13.5%)	0.96
Invasive mechanical ventilation	7 (9.5%)	7 (13.5%)	0.68
Renal replacement therapy	6 (8.1%)	4 (7.7%)	~1

[1]Mood's test for median values, Chi-squared test for categorical data

[2]See eTable 3 for ICD-10 code lists

[3]Bisoprolol, Atenolol, Propranolol, Carvedilol.

eTable 3. International Classification of Diseases 10th edition (ICD-10) codes used to identify comorbidities.

Diagnosis	ICD-10 codes	Description
**Heart disease**	I20	Angina pectoris
	I21	Acute myocardial infarction
	I22	Subsequent myocardial infarction
	I23	Certain current complications following acute myocardial infarction
	I24	Other acute ischaemic heart diseases
	I25	Chronic ischaemic heart disease
	I34	Nonrheumatic mitral valve disorders
	I35	Nonrheumatic aortic valve disorders
	I36	Nonrheumatic tricuspid valve disorders
	I37	Pulmonary valve disorders
	I42	Cardiomyopathy
	I43	Cardiomyopathy in diseases classified elsewhere
	I44	Atrioventricular and left bundle-branch block
	I50	Heart failure
**Hypertension**	I10	Essential hypertension
	I11	Hypertensive heart disease
	I12	Hypertensive renal disease
	I13	Hypertensive heart and renal disease
	I15	Secondary hypertension
**Diabetes**	E10	Type 1 diabetes mellitus
	E11	Type 2 diabetes mellitus
	E12	Malnutrition-related diabetes mellitus
	E13	Other specified diabetes mellitus
	E14	Other unspecified diabetes mellitus
**Endocrine disease (other than diabetes)**	E00	Congenital iodine-deficiency syndrome
	E01	Iodine-deficiency-related thyroid disorders and allied conditions

	E02	Subclinical iodine-deficiency hypothyroidism
	E03	Other hypothyroidism
	E04	Other nontoxic goitre
	E05	Thyrotoxicosis
	E06	Thyroiditis
	E07	Other disorders of thyroid
	E20	Hypoparathyroidism
	E21	Hyperparathyroidism and other disorders of parathyroid gland
	E22	Hyperfunction of pituitary gland
	E23	Hypofunction and other disorders of pituitary gland
	E24	Cushing syndrome
	E25	Adrenogenital disorders
	E26	Hyperaldosteronism
	E27	Other disorders of adrenal gland
	E28	Ovarian dysfunction
	E29	Testicular dysfunction
	E30	Disorders of puberty, not elsewhere classified
	E31	Polyglandular dysfunction
	E32	Diseases of thymus
	E34	Other endocrine disorders
	E35	Disorders of endocrine glands in diseases classified elsewhere
	E89.0	Postprocedural hypothyroidism
	E89.1	Postprocedural hypoinsulinemia
	E89.2	Postprocedural hypoparathyroidism
	E89.3	Postprocedural hypopituitarism
	E89.4	Postprocedural ovarian failure
	E89.5	Postprocedural testicular hypofunction
	E89.6	Postprocedural adrenocortical (-medullary) hypofunction
**Stroke**	I63	Cerebral infarction
	I65	Occlusion and stenosis of precerebral arteries, not

		resulting in cerebral infarction
	I66	Occlusion and stenosis of cerebral arteries, not resulting in cerebral infarction
**Dementia**	F01	Vascular dementia
	F02	Dementia in other diseases classified elsewhere
	F03	Unspecified dementia
	G30, G31	Alzheimer disease & Other degenerative diseases of nervous system, not elsewhere classified
	F10.27	Alcohol dependence, with alcohol-induced persisting dementia
	F10.97	Alcohol use, unspecified with alcohol-induced persisting dementia
	F19.97	Other psychoactive substance use, unspecified with psychoactive substance-induced persisting dementia
**Asthma**	J45	Asthma
**Respiratory disease (other than asthma)**	I27	Other pulmonary heart diseases
	J6*-J7*	Lung diseases due to external agents
	J41	Simple and mucopurulent chronic bronchitis
	J42	Unspecified chronic bronchitis
	J43	Emphysema
	J44	Other chronic obstructive pulmonary disease
	J47	Bronchiectasis
**Chronic kidney disease**	N18.1-N18.5	Chronic kidney disease stage 1-5
	N18.9	Chronic kidney disease, unspecified
	I13	Hypertensive and renal disease
**Chronic liver disease**	K70	Alcoholic liver disease
	K71	Toxic liver disease
	K72	Hepatic failure, not elsewhere classified
	K73	Chronic hepatitis, not elsewhere classified
	K74	Fibrosis and cirrhosis of the liver
	K75	Other inflammatory diseases of the liver
	K76	Other diseases of the liver
	K77	Liver disorders in disease classified elsewhere

**Malignancy non-haematological**	C0	Malignant neoplasm of lip
	C1	Malignant neoplasm of base of tongue
	C2	Malignant neoplasm of other unspecified parts of tongue
	C3	Malignant neoplasm of gum
	C4	Malignant neoplasm of floor of mouth
	C5	Malignant neoplasm of palate
	C6	Malignant neoplasm of other and unspecified parts of mouth
	C7	Malignant neoplasm of parotid gland
**Malignancy haematological**	C8	Malignant neoplasm of other and unspecified major salivary glands
	C9	Malignant neoplasm of tonsil
**Immunocompromised**	D80	Immunodeficiency with predominantly antibody defects
	D81	Combined immunodeficiencies
	D82	Immunodeficiency associated with other major defects
	D83	Common variable immunodeficiency
	D84	Other immunodeficiencies

eFigure 1. Scatter plots of paired aldosterone and renin results with linear regression line, with the two extreme outliers shown included.

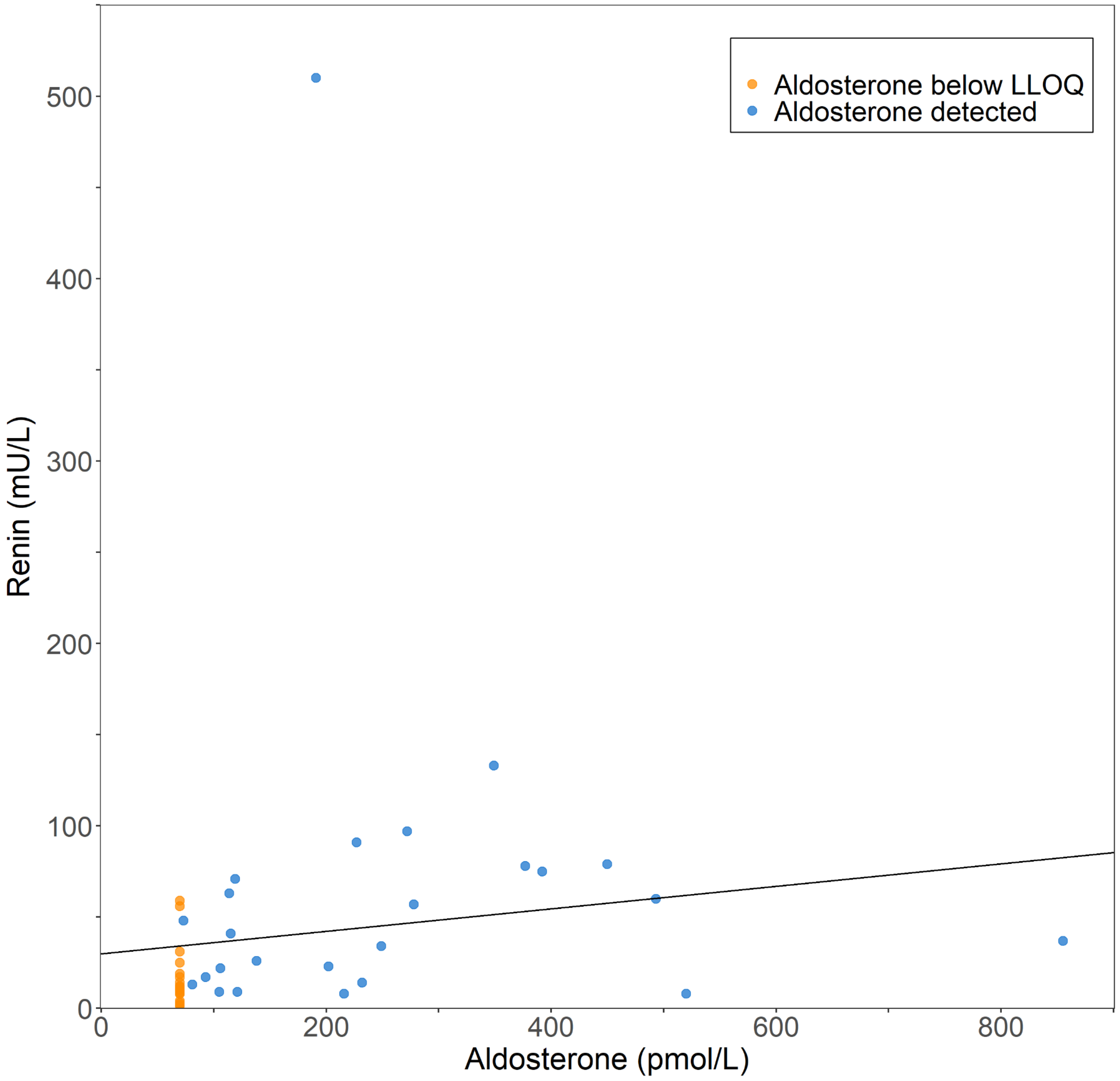

eFigure 2. Kaplan-Meier survival plot for 28-day survival after first positive test, stratified by whether the first aldosterone after the positive test was above or below the LLOQ.

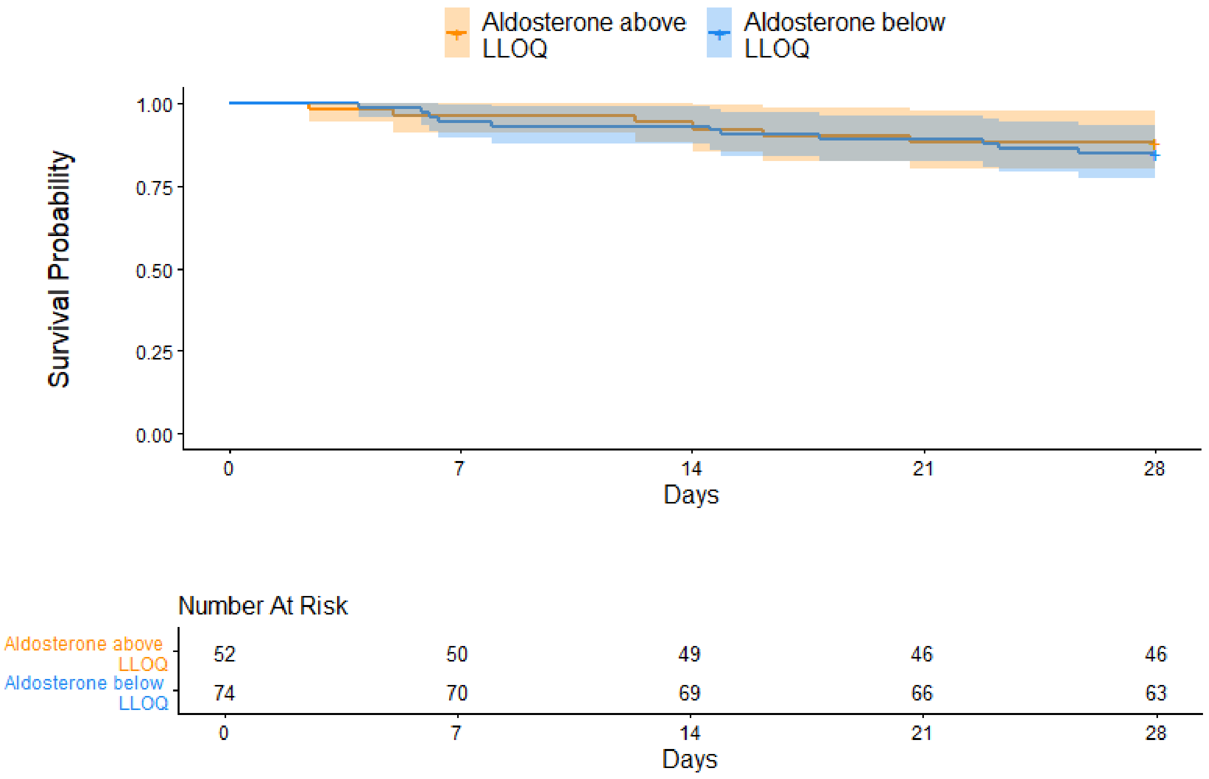

eFigure 3. Association plots of the LCMSMS aldosterone measurements (y-axis) and **(A)** eGFR **(B)** creatinine clearance (Cockcroft-Gault). The solid lines indicate (robust) linear regression.

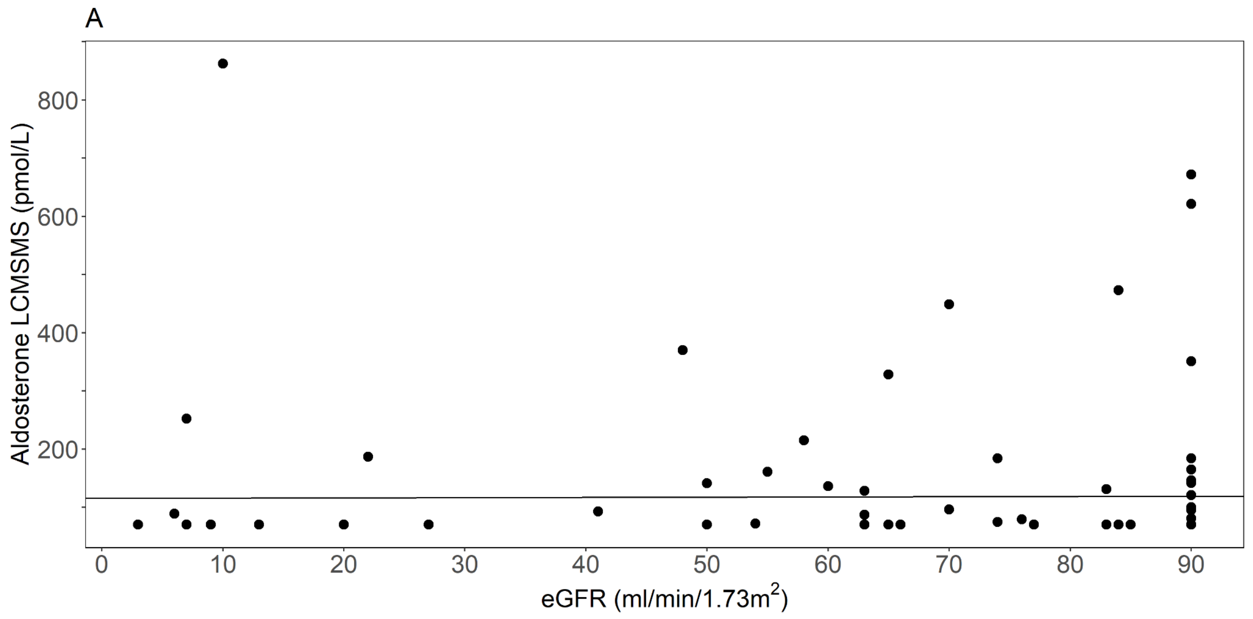

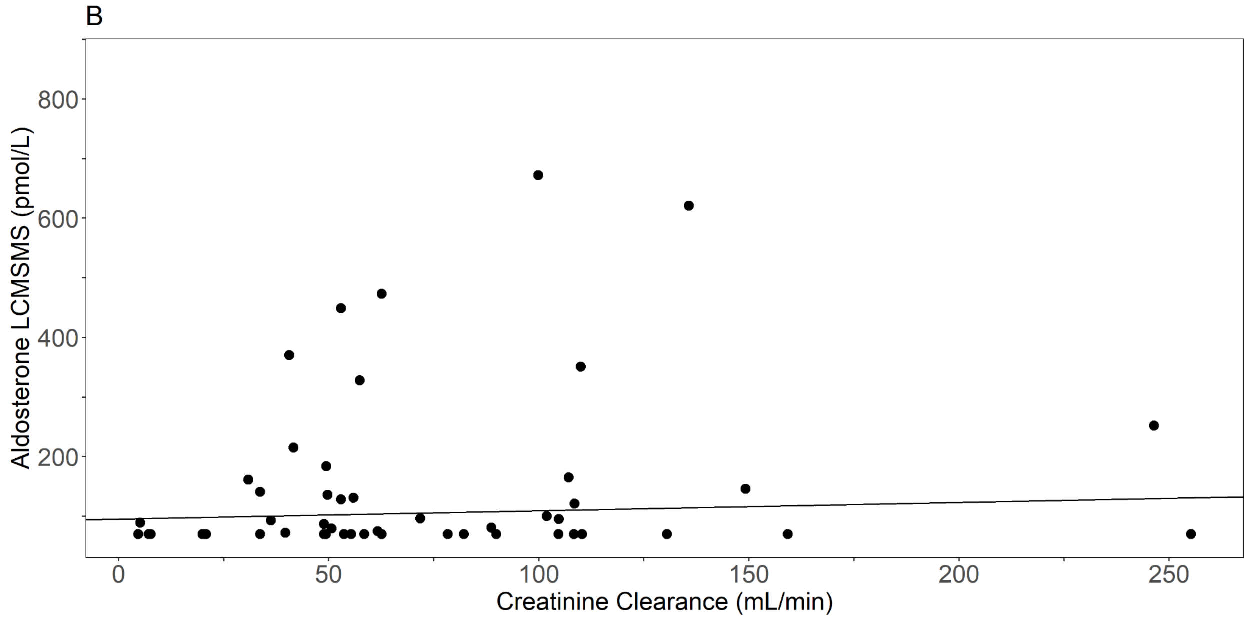

eFigure 4. Bland-Altman plot showing the average of the aldosterone concentration measured by CLIA and LCMSMS on the x-axis and the raw difference between methods on the y-axis.

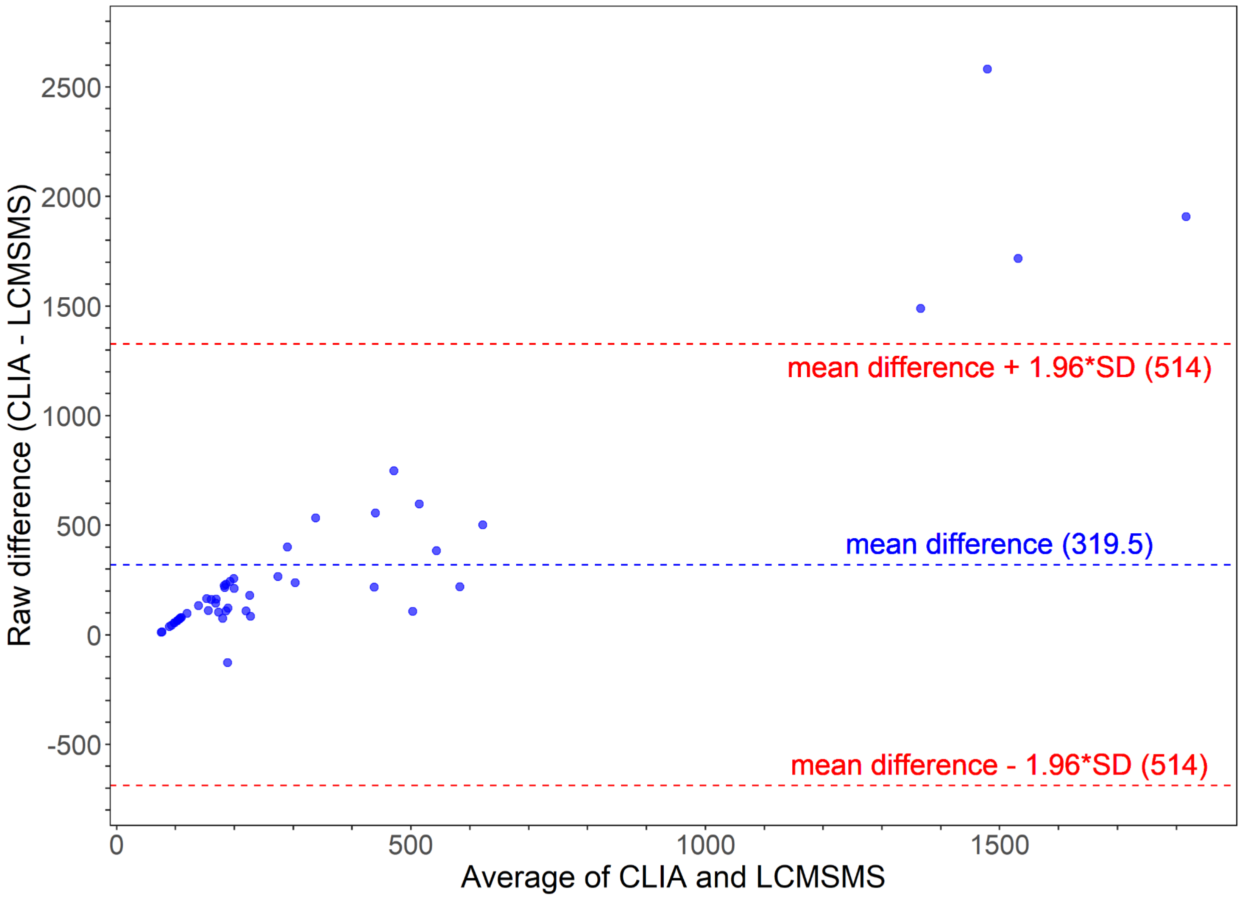

eFigure 5. Association plots of the difference between CLIA and LCMSMS aldosterone measurements (y-axis) and **(A)** eGFR **(B)** creatinine clearance (Cockcroft-Gault) **(C)** bilirubin **(D)** ALT and **(E)** Cortisol. The solid lines indicate (robust) linear regression.

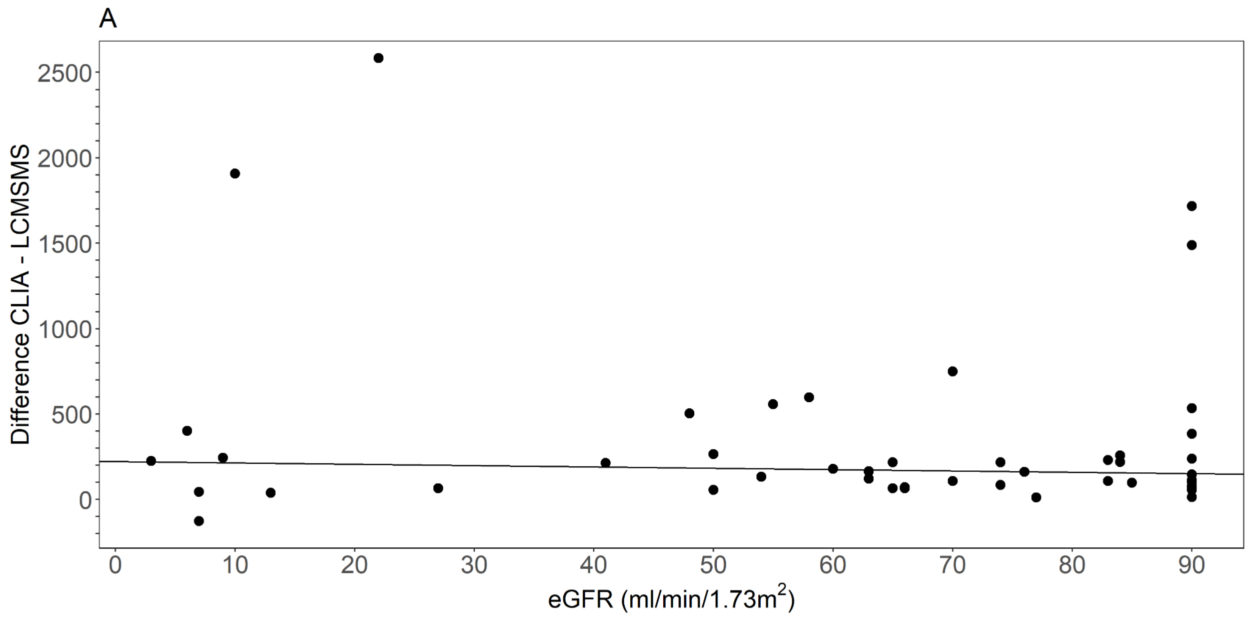

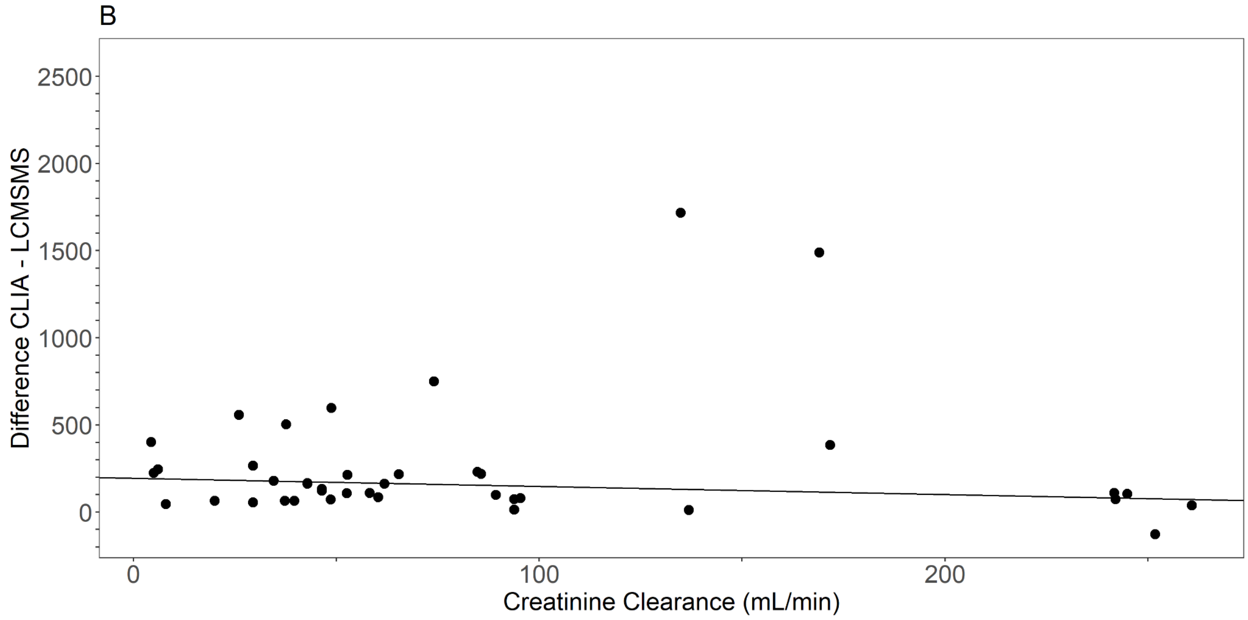

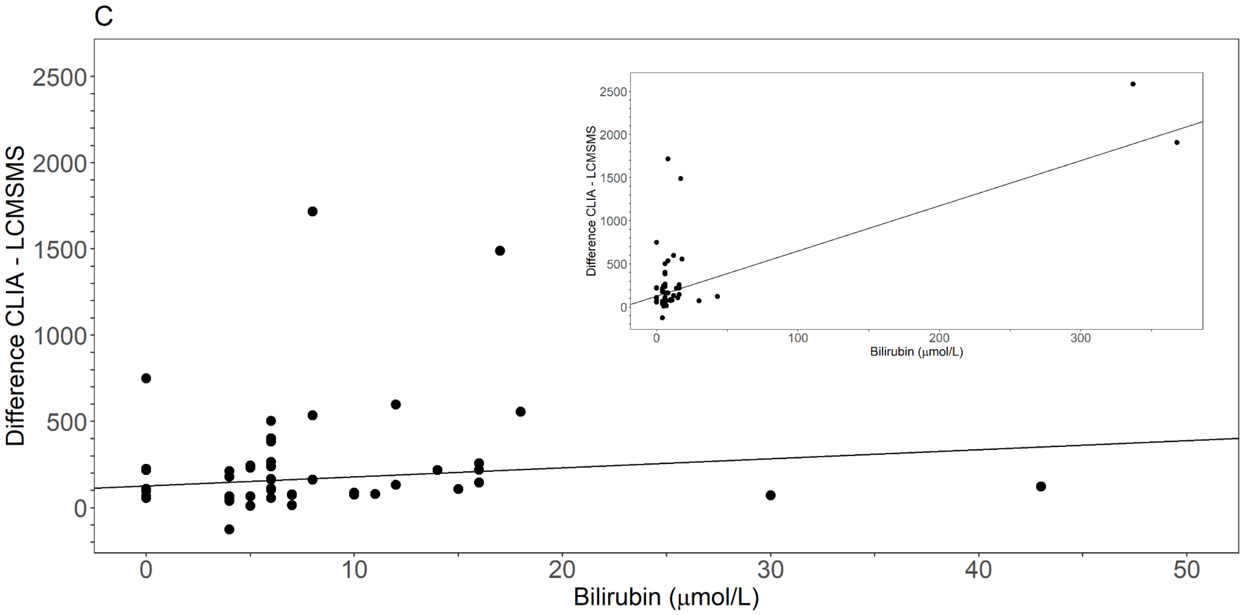
C
Difference CLIA - LCMSMS
Bilirubin (µmol/L)
2500
2000
1500
1000
500
0
0
10
20
30
40
50
Difference CLIA - LCMSMS
Bilirubin (µmol/L)
100
200
300

D

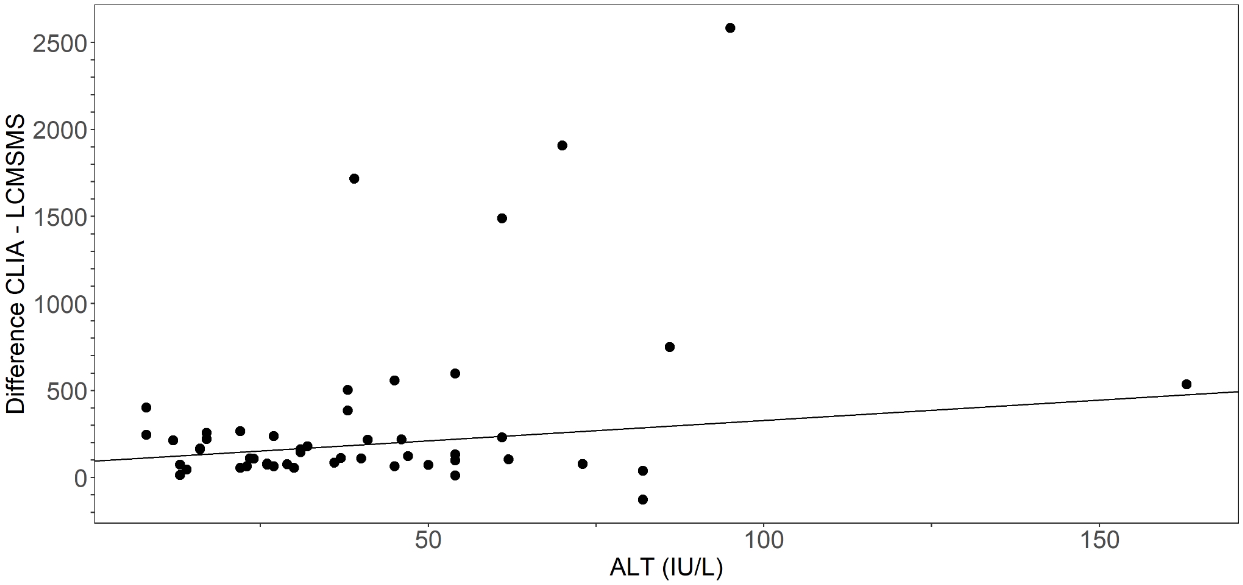

Difference CLIA - LCMSMS
2500
2000
1500
1000
500
0
50
100
150
ALT (IU/L)

E

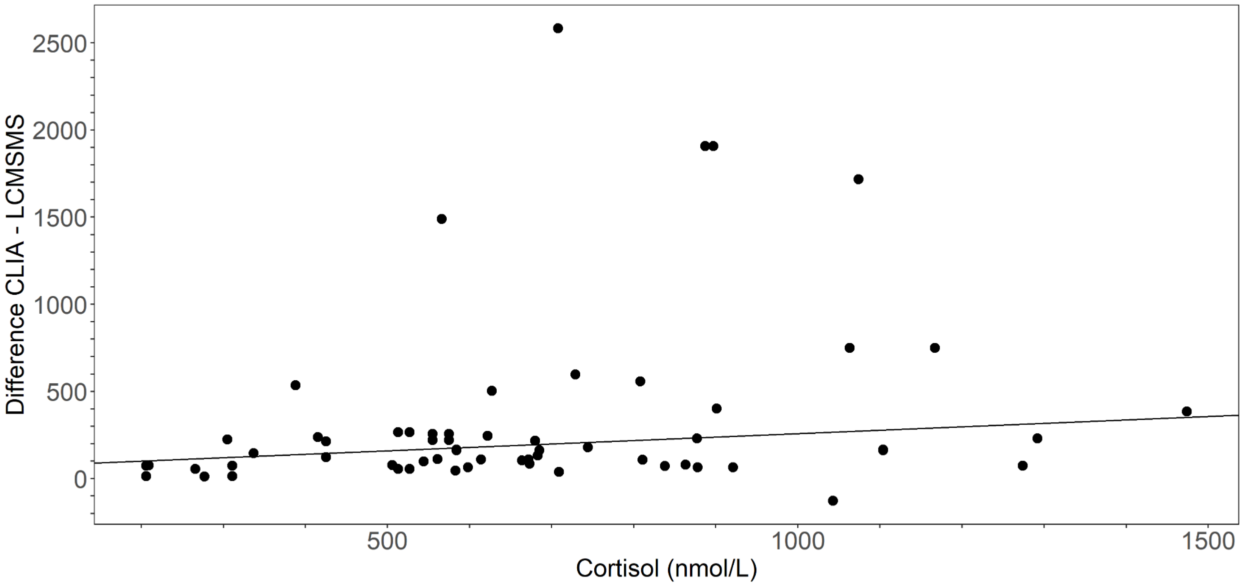

Difference CLIA - LCMSMS
2500
2000
1500
1000
500
0
500
1000
1500
Cortisol (nmol/L)

eFigure 6. Comparison of LCMSMS (x-axis) and CLIA aldosterone (y-axis) results, with solid lines indicating Passing-Bablok regression lines, solvent extracted (red) and non-extracted (gray). The dotted line indicates the y=x identity line.

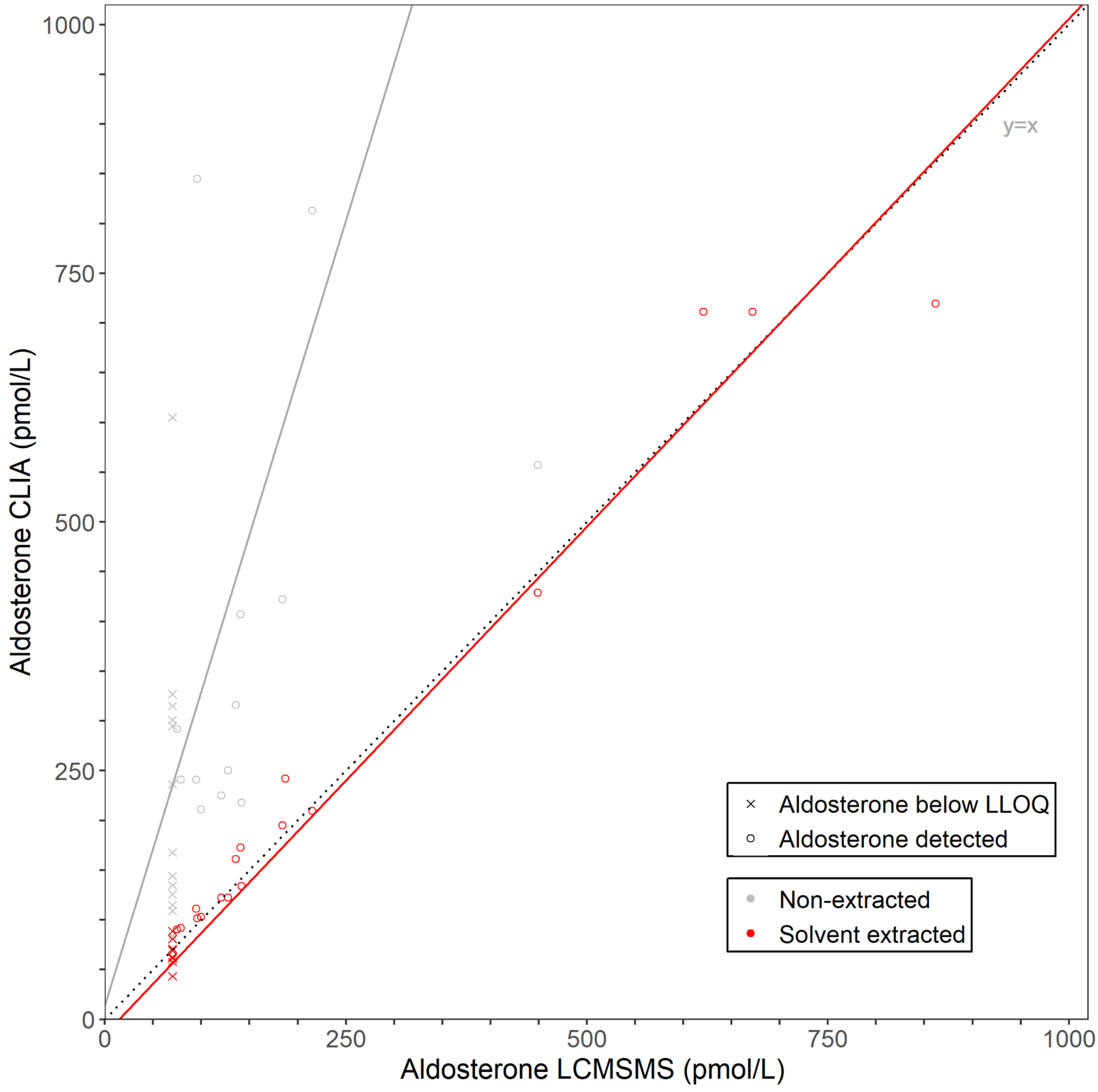

Supplement: Supplementary material [file EMS153893-supplement-Supplementary_material.pdf]
